# Dermoscopy of venous lake on the lips: A comparative study with labial melanotic macule

**DOI:** 10.1371/journal.pone.0206768

**Published:** 2018-10-31

**Authors:** Ji Su Lee, Je-Ho Mun

**Affiliations:** 1 Department of Dermatology, Seoul National University College of Medicine, Seoul, Korea; 2 Institute of Human-Environment Interface Biology, Seoul National University, Seoul, Korea; University of Queensland Diamantina Institute, AUSTRALIA

## Abstract

**Background:**

Venous lake (VL) is a common vascular tumor occurring on the lips in the elderly. VL is sometimes difficult to distinguish from melanotic lesions such as labial melanotic macule (LMM) or oral malignant melanoma. However, the dermoscopic features of VL have not been sufficiently established in the literature.

**Objective:**

This study was aimed at investigating the dermoscopic features of VL on the lips, and to compare the dermoscopic features of VL with those of LMM.

**Methods:**

We retrospectively investigated the dermoscopic findings of histopathologically proven cases of VL and LMM.

**Results:**

The structureless pattern (78.6%) and globules/clods (42.9%) were the common patterns in VL cases. Purple was the most frequent color (78.6%), followed by red (42.9%) and blue (42.9%). The structureless pattern (p = 0.003) and the colors purple (p = 0.000), red (p = 0.003), and blue (p = 0.018) were significantly more common in VL than in LMM. In contrast, lines (p = 0.000) and dots (p = 0.044) as patterns, and brown (p = 0.000) and gray (p = 0.044) colors were significantly more frequent in LMM. White structures were more common in VL than in LMM (p = 0.001).

**Conclusion:**

Structureless patterns or globules/clods with purple, red, or blue coloration can be useful findings when differentiating VLs from LMM on dermoscopy. Therefore, dermoscopic evaluation is a helpful noninvasive ancillary tool in the diagnosis of VL.

## Introduction

Venous lake (VL) is a common benign vascular tumor that is also known as senile hemangioma and phlebectasia.[1] VL typically presents as soft, compressible, dark-blue to violaceous papules or nodules on the head and neck, especially on the lower lips of elderly people.[2–4] Although the lesion is usually asymptomatic, it is cosmetically distressful and occasionally bleeds or becomes painful.[4] Histopathologically, VL shows 1 to several dilated vascular spaces with erythrocytes and occasionally thrombi lying on the irregularly arranged fibrous stroma.[2, 4] Usually, severe solar elastosis is evident in adjacent dermis.[3]

VL is easy to diagnose in cases with a typical presentation. However, in some cases, VL on the lips can be confused with pigmented lesions such as labial melanotic macule (LMM) or oral malignant melanoma (OMM).[1, 3–5] Therefore, it is important to differentiate VL from LMM because the treatment modalities are different for each disease; VL is a vascular tumor, whereas LMM is a melanotic lesion.[4, 6] Dermoscopy can be a useful non-invasive diagnostic tool for various pigmented and non-pigmented tumors.[7–12] However, although the dermoscopic patterns of LMM have been sufficiently reported,[13–16] the dermoscopic features of VL have been scarcely described in the literature.[17, 18] This study was aimed at investigating the dermoscopic features of histopathologically confirmed VL on the lips and to compare them with those of LMM.

## Materials and methods

**Fig 1:**
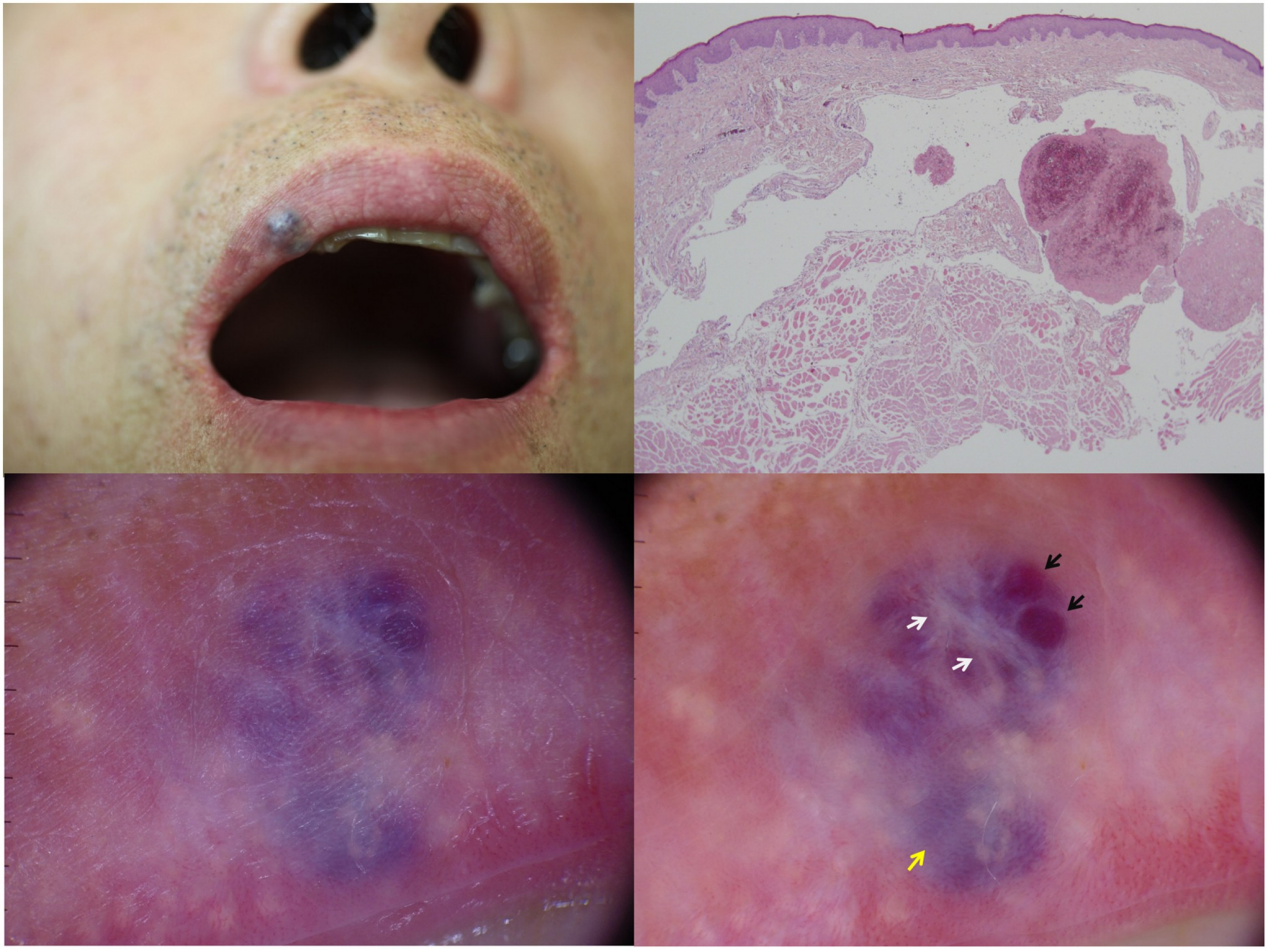
Clinical, dermoscopic, and histopathologic pictures of venous lake on the lips. (a) A dark blue papule located on the upper lip. (b) Histopathologic findings showing dilated vascular spaces and thrombi located within the vascular spaces in the upper dermis. (hematoxylin and eosin, ×40). (c) Nonpolarized dermoscopic images of venous lake taken without the use of interface fluid. (d) Polarized dermoscopic images of venous lake. Polarized dermoscopy enables better observation of color and white structures than nonpolarized dermoscopy. Venous lake shows globules/clods (black arrows), a structureless pattern (yellow arrow), and white structures (white arrows).

Patients with a histopathologically confirmed VL diagnosis and available clinical and dermoscopic images, who attended Seoul National University Hospital, Seoul, South Korea, between November 2015 and June 2018, were enrolled in our study. The following demographic information was obtained from medical records: age; sex; and lesion location, duration, and number. Dermoscopic images of each lesion were obtained using DermLiteDL3 (3Gen, Dana Point, CA, USA) with ×10 magnification. The polarized dermoscopic mode enables better observation of the patterns and colors of VL lesions than does the nonpolarized dermoscopic mode without the use of interface fluid (Fig 1).

Therefore, we analyzed polarized dermoscopic images. Pressure was not applied to VL to avoid collapse of vessels. Dermoscopic images were independently evaluated by 2 dermatologists (Mun and Lee), with final determinations made by consensus. On the basis of the recent consensus dermoscopic terminologies of the International Society of Dermoscopy,[19] the following dermoscopic features were evaluated: patterns (structureless, globules/clods, dots, lines, and circles), colors (purple, red, blue, gray, black, and brown), presence of white structures (shiny white lines or shiny white structureless area), and presence and morphologic details of vascular structures. The number of patterns and colors present in each lesion were counted. To rule out the influence of normal vascularity of the lip mucosa, vessels were considered only when they were prominent relative to the vessels of the adjacent nonlesional mucosa. In the second part of the study, dermoscopic images of histopathologically proven cases of LMM during the same period were investigated for a comparative analysis.

Differences between the 2 groups were determined using the chi-square test or Fisher’s exact test for categorical values, and the Student’s t-test or Mann-Whitney U-test for continuous variables. Values of p < 0.05 were considered statistically significant. Interobserver agreement in the assessment of dermoscopic features was evaluated using Cohen’s kappa. Statistical analysis was performed with SPSS version 23.0 software (IBM Corp., Armonk, NY, USA).

This study was approved by the Institutional Review Board at Seoul National University, and was conducted with strict adherence to the principles of the Declaration of Helsinki.

## Results

A total of 14 cases of VL were evaluated. The mean patient age at the diagnosis of VL was 61.4 years (range, 21–89 years), and the sex ratio was 6:1 (women to men). The mean lesion duration was 5.9 years (range, 0.4–30 years). The lower lips were involved in most cases (85.7%, 12 of 14), and 1 patient had VL on both the lower and upper lips. Most patients (78.6%) had a single lesion. The demographic findings of VL and LMM cases are summarized in Table 1. There were no statistically significant differences in age; sex ratio; and lesion duration, location, and number between VL and LMM.

**Table 1:** Demographic findings of cases of venous lakes and labial melanotic macules on the lips.

	Venous lake (n = 14)	Labial melanotic macule (n = 16)	p-Value
**Age (years)**	61.4 (21–89)	53 (19–81)	0.162
**Women/men**	12/2	10/6	0.151
**Lesion duration (years)**	5.9 (0.4–30)^a^	1.6 (0.3–5)^b^	0.667
**Location (%)**			0.402
**Upper lip**	2 (14.3)	1 (6.2)	
**Lower lip**	11 (78.6)	15 (93.8)	
**Both lips**	1 (7.1)	0 (0)	
**Lesion number (%)**			0.142
**Single**	11 (78.6)	8 (50)	
**Multiple (≥2)**	3 (21.4)	8 (50)	

^a^Data available in 11 cases.

^b^Data available in 14 cases.

In the analysis of dermoscopic features of VL, the structureless pattern (78.6%) was the most common pattern, followed by globules/clods (42.9%). Most cases presented with a single pattern (85.7%) and the presence of 2 patterns was observed in 3 cases (21.4%). The observed colors, in order of frequency, were purple (78.6%), red (42.9%), and blue (42.9%). White structures were observed in 64.3% of cases. Vascular structures were seen in 28.6% of cases, and all of them were linear-irregular vessels. The interobserver agreement was good to excellent (Cohen’s kappa, 0.77–1.00) for all variables of VL.

**Fig 2:**
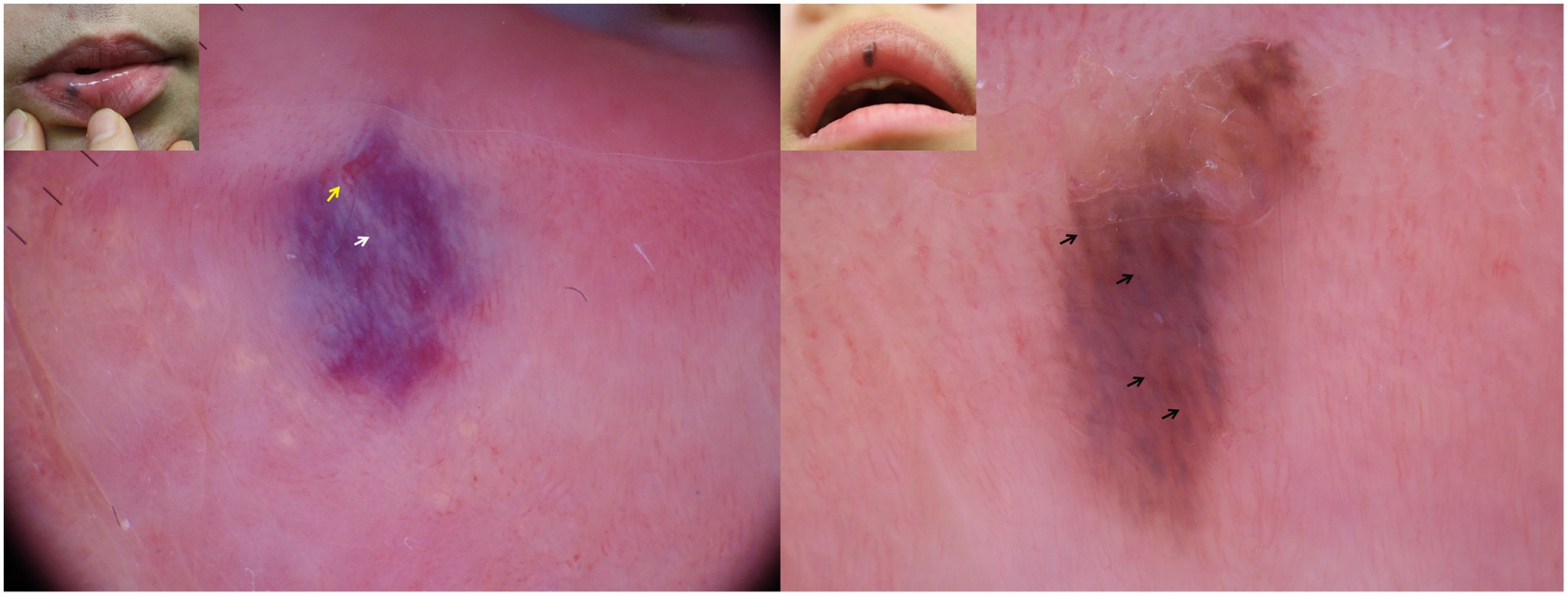
Dermoscopic findings and clinical images (insets) of venous lake on the lips and labial melanotic macules. (a) Venous lake on the lower lip showing a purple to red structureless pattern with white structures (white arrows) and linear-irregular vessels (yellow arrows). (b) Labial melanotic macule on the upper lip composed of brown parallel lines (black arrows).

Concerning the dermoscopic features of 16 cases of LMM (Table 2 and Fig 2), the most common pattern was lines (81.3%).

**Table 2:** Dermoscopic patterns and colors observed in venous lake and labial melanotic macules on the lips.

	Venous lake (n = 14)	Labial melanotic macule (n = 16)	p-Value
**Patterns, type**			
**Structureless**	11 (78.6)	3 (18.6)	**0.003**
**Globules/clods**	6 (42.9)	2 (12.5)	0.134
**Dots**	0 (0)	4 (25)	**0.044**
**Lines**	0 (0)	13 (81.3)	**<0.001**
**Circles**	0 (0)	2 (12.5)	0.171
**Patterns, number**			0.166
**1**	11 (78.6)	9 (56.2)	
**2**	3 (21.4)	6 (37.5)	
**3**	0 (0)	1 (6.3)	
**Colors**			
**Purple**	11 (78.6)	0 (0)	**<0.001**
**Red**	6 (42.9)	0 (0)	**0.003**
**Blue**	6 (42.9)	1 (6.3)	**0.018**
**Gray**	0 (0)	4 (25)	**0.044**
**Black**	0 (0)	1 (6.3)	0.341
**Brown**	0 (0)	15 (93.8)	**<0.001**
**Colors, number**			0.131
**1**	5 (35.7)	11 (68.8)	
**2**	9 (64.3)	5 (31.2)	
**White structures**	9 (64.3)	1 (6.3)	**0.001**
**Vascular structures**	4 (28.6)	1 (6.3)	0.102

Bold values are statistically significant (p < 0.05).

The most common color in LMM was brown (93.8%), followed by gray (25%), blue (6.3%), and black (6.3%). Statistical analysis revealed that the structureless pattern was more frequent in VL (p = 0.003). Conversely, lines (p = 0.000) and dots (p = 0.044) were more common patterns in LMM. Purple (p = 0.000), red (p = 0.003), and blue (p = 0.018) lesions were more frequently observed in VL; in contrast, brown (p = 0.000) and gray (p = 0.044) lesions were more often detected in LMM. White structures were detected more commonly in VL than in LMM (p = 0.001). The number of total patterns or colors showed no statistically significant difference between VL and LMM.

## Discussion

VL is believed to occur as a result of solar damage to the vascular adventitia and dermal elastic fibers.[3, 4] The preponderance of occurrence in sun-exposed areas including the face, neck, helix of the ears, and lips in elderly patients substantiates this explanation.[4] In line with previous findings, the mean age of patients was 61.7 years, and the lower lip, which is prone to sun damage, was involved in 85.7% (12 of 14) of patients in the present study.[2, 20, 21]

To our knowledge, this is the first study with an in-depth analysis of the dermoscopic features of VL. Our results showed the structureless pattern (57.1%), globules/clods (21.4%), and the combination of these 2 patterns (21.4%) in VLs. The histological correlation of the structureless pattern and globules/clods seem to be associated with dilated vascular spaces.[22] In terms of color, purple (78.6%) was the most common, followed by red (42.9%) and blue (42.9%).

Owing to their potential dark color and nodularity, VLs on the lips may appear similar to LMM or OMM.[4] However, by using dermoscopy, LMM can be easily differentiated from VL. In line with previous findings,[13–16] our data showed that LMMs mainly consisted of brown coloration with parallel lines, dots, or circles. The statistical findings between the 2 entities can be clearly differentiated when using dermoscopy.

Blum *et al*. proposed that the combination of blue, gray, or white color with structureless zones is a strong indicator for malignancy of mucosal pigmented lesions.[16] However, VL should be an exception to the rule because, in our data, structureless patterns, white structures, and blue color were not uncommon in VL. OMMs are usually composed of more than 2 colors; brown and gray are the most common, followed by blue and white.[16] In contrast, in VL, purple was the most frequent color, and there was no case that presented a brown or gray color. Furthermore, a multicomponent pattern (presence of 3 or more patterns in the same lesion), atypical vessels, and asymmetry of overall structures are other important dermoscopic findings of OMM (Table 3).[15, 16, 23–26]

**Table 3:** Clinical and dermoscopic features of venous lake, labial melanotic macule, and oral malignant melanoma.

	Clinical features	Dermoscopic features
**Venous lake**	∙ Mean onset age of 60–65 years ∙ Soft, compressible, dark blue to purple, flat or slightly dome-shaped papules or nodules ∙ Lower lip > upper lip	∙ Structureless patterns or globules/clods with purple, red, or blue coloration
**Labial melanotic macule**	∙ Usually occurs in 4^th^ or 5^th^ decade of life, F > M ∙ Usually single, discrete, brown to black macule ∙ Lower lip > upper lip	∙ Brown coloration with parallel lines, dots, or circles; sometimes with gray coloration
**Oral malignant melanoma**	∙ Rare (0.1–8% of all melanomas) ∙ Mainly occurs in the 4^th^–7^th^ decades of life, mean onset age of 55–60 years, M > F (2:1) ∙ Solitary, brown to black macule, patch or nodule often showing asymmetry and irregular borders ∙ 5–35% of all cases are amelanotic ∙ Ulceration occurs up to 1/3 of cases	∙ Usually composed of >2 colors (brown, gray, blue, and white) ∙ Asymmetry of overall structures, multicomponent pattern, atypical pigmentation network, blue-whitish veil, ulceration, irregularly distributed dots/clods, and atypical vessels

F, female; M, male.

As VLs do not spontaneously involute, treatment is often required to improve cosmetic appearance and prevent recurrent bleeding.[6] Various treatments have been reported, including surgical excision, cryosurgery, sclerotherapy, electrocoagulation, and laser- and light-based modalities.[4, 6] In particular, pulsed dye laser and long-pulsed (1064-nm) neodymium:yttrium‐aluminum‐garnet (Nd:YAG) laser have advantages of minimal discomfort during the procedure, less posttreatment scarring, and high efficacy.[4] However, for treating LMM, Q-switched Nd:YAG laser at 532-nm wavelength is usually used.[27] Therefore, differentiation of VL and LMM is crucial in selecting the appropriate laser modalities and parameters.

In conclusion, structureless patterns or globules/clods with purple, red, or blue coloration can be useful when differentiating VLs from LMM in dermoscopy. Dermoscopic evaluation can be a helpful noninvasive ancillary diagnostic tool in the diagnosis of VL.

## Supporting information

S1 Table Clinical and dermoscopic features of patients with venous lake. (DOCX)

S2 Table Clinical and dermoscopic features of patients with labial melanotic macule. (DOCX)
