# Supplementary material for: Dermoscopy of venous lake on the lips: A comparative study with labial melanotic macule
Source: PLoS One. 2018 Oct 31;13(10):e0206768. doi: 10.1371/journal.pone.0206768 (PMC6209377; doi:10.1371/journal.pone.0206768)
Supplement: S1 Table — (DOCX) [file pone.0206768.s001.docx]

**Supplemental data 1. Clinical and dermoscopic features of patients with venous lake**

|  | **Clinical features** | | | | | **Dermoscopic features** | | | | | | | | | | | | | |
| --- | --- | --- | --- | --- | --- | --- | --- | --- | --- | --- | --- | --- | --- | --- | --- | --- | --- | --- | --- |
|  |  |  |  |  |  | **Pattern** | | | | | **Color** | | | | | | **White structure** | **Vascular structures** | |
|  |  | Age | Location(1=upper,2=lower,3=both) | Lesion number (1=single, 2=multiple) | Duration(years) | Structureless | Globules/clods | Lines | Circles | Dots | Blue | Red | Purple | Black | Brown | Others |  | Presence | Morphology |
| 1 | F | 70 | 2 | 1 | N/A | + | + |  |  |  | + |  | + |  |  |  | + |  |  |
| 2 | F | 82 | 2 | 1 | 0.5 | + |  |  |  |  | + |  | + |  |  |  | + | + | LI |
| 3 | F | 68 | 2 | 1 | 3 | + | + |  |  |  | + |  |  |  |  |  | + |  |  |
| 4 | F | 61 | 1 | 1 | 10 |  | + |  |  |  |  |  | + |  |  |  | + |  |  |
| 5 | F | 64 | 2 | 1 | N/A | + |  |  |  |  | + |  |  |  |  |  |  |  |  |
| 6 | M | 66 | 1 | 1 | 30 | + | + |  |  |  |  | + | + |  |  |  | + |  |  |
| 7 | F | 21 | 2 | 1 | 2.5 | + |  |  |  |  | + | + |  |  |  |  | + | + | LI |
| 8 | F | 47 | 2 | 1 | 1 | + |  |  |  |  | + |  | + |  |  |  |  |  |  |
| 9 | F | 78 | 2 | 1 | 6.5 | + |  |  |  |  |  | + | + |  |  |  |  |  |  |
| 10 | M | 63 | 2 | 2 | 3 |  | + |  |  |  |  | + | + |  |  |  | + |  |  |
| 11 | F | 51 | 2 | 1 | 1.5 | + |  |  |  |  |  | + | + |  |  |  | + | + | LI |
| 12 | F | 53 | 2 | 1 | 0.42 | + |  |  |  |  |  |  | + |  |  |  |  |  |  |
| 13 | F | 69 | 2 | 2 | 6 |  | + |  |  |  |  |  | + |  |  |  | + | + | LI |
| 14 | F | 66 | 3 | 2 | N/A | + |  |  |  |  |  | + | + |  |  |  |  |  |  |

LI, linear irregular
