# Supplementary material for: Dermoscopy of venous lake on the lips: A comparative study with labial melanotic macule
Source: PLoS One. 2018 Oct 31;13(10):e0206768. doi: 10.1371/journal.pone.0206768 (PMC6209377; doi:10.1371/journal.pone.0206768)
Supplement: S2 Table — (DOCX) [file pone.0206768.s002.docx]

**Supplemental data 2. Clinical and dermoscopic features of patients with labial melanotic macule**

|  | **Clinical features** | | | | | **Dermoscopic features** | | | | | | | | | | | | | |
| --- | --- | --- | --- | --- | --- | --- | --- | --- | --- | --- | --- | --- | --- | --- | --- | --- | --- | --- | --- |
|  |  |  |  |  |  | **Pattern** | | | | | **Color** | | | | | | **White structure** | **Vascular structures** | |
|  |  | Age | Location(1=upper,2=lower,3=both) | Lesion number (1=single, 2=multiple) | Duration(years) | Structureless | Globules/clods | Lines | Circles | Dots | Blue | Red | Purple | Black | Brown | Grey |  | Presence | Morphology |
| 1 | F | 59 | 2 | 1 | 0.5 |  |  | + |  |  |  |  |  |  | + |  |  |  |  |
| 2 | F | 55 | 2 | 1 | 0.4166667 |  |  | + |  |  |  |  |  |  | + | + |  |  |  |
| 3 | F | 22 | 2 | 2 | 0.25 | + |  |  |  |  |  |  |  |  | + |  |  |  |  |
| 4 | F | 81 | 2 | 2 | N/A |  |  | + |  | + |  |  |  |  | + |  |  |  |  |
| 5 | F | 45 | 2 | 1 | 1 |  |  | + |  |  |  |  |  |  | + |  |  | + | LI |
| 6 | F | 62 | 2 | 1 | 1 |  |  | + |  |  |  |  |  |  | + | + |  |  |  |
| 7 | M | 63 | 2 | 2 | 1 |  |  | + |  | + |  |  |  |  | + | + |  |  |  |
| 8 | F | 43 | 2 | 1 | 0.3333333 |  | + |  |  |  |  |  |  |  | + |  |  |  |  |
| 9 | F | 19 | 2 | 2 | 3 | + |  |  |  |  |  |  |  |  | + |  |  |  |  |
| 10 | M | 45 | 2 | 1 | 4.5 | + |  | + |  |  | + |  |  | + |  |  | + |  |  |
| 11 | F | 69 | 2 | 2 | N/A |  |  | + |  |  |  |  |  |  | + |  |  |  |  |
| 12 | M | 48 | 2 | 1 | 1 |  |  | + | + |  |  |  |  |  | + |  |  |  |  |
| 13 | M | 63 | 2 | 2 | 5 |  |  | + |  | + |  |  |  |  | + |  |  |  |  |
| 14 | M | 71 | 2 | 2 | 0.75 |  |  | + |  |  |  |  |  |  | + |  |  |  |  |
| 15 | M | 62 | 2 | 1 | 1 |  |  | + | + | + |  |  |  |  | + | + |  |  |  |
| 16 | F | 39 | 1 | 2 | 3 |  | + | + |  |  |  |  |  |  | + |  |  |  |  |

LI, linear irregular
